# High-Intensity Laser Therapy Versus Extracorporeal Shockwave Therapy for Lateral Elbow Tendinopathy: A Systematic Review and Meta-Analysis

**DOI:** 10.3390/bioengineering13020155

**Published:** 2026-01-28

**Authors:** Pei-Ching Wu, Dung-Huan Liu, Yang-Shao Cheng, Chih-Sheng Lin, Fu-An Yang

**Affiliations:** 1 Department of Chinese Medicine, China Medical University Hospital, Taichung City 404327, Taiwan; 036619@tool.caaumed.org.tw; 2 College of Chinese Medicine, China Medical University, Taichung City 404333, Taiwan; 3 Graduate Degree Program of Biomedical Science and Engineering, National Yang Ming Chiao Tung University, Hsinchu City 300193, Taiwan; 035345@tool.caaumed.org.tw (D.-H.L.); lincs@mail.nctu.edu.tw (C.-S.L.); 4 Department of Physical Medicine and Rehabilitation, Taichung Municipal Geriatric Rehabilitation General Hospital Managed by China Medical University, China Medical University, Taichung City 406004, Taiwan; 5 Department of Physical Medicine and Rehabilitation, China Medical University Hospital, Taichung City 404327, Taiwan; hsnu128439@gmail.com; 6 Department of Physical Therapy, Graduate Institute of Rehabilitation Science, China Medical University, Taichung City 406040, Taiwan; 7 Department of Education, China Medical University, Taichung City 404333, Taiwan; 8 Department of Biological Science and Technology, National Yang Ming Chiao Tung University, Hsinchu City 300193, Taiwan; 9 Center for Intelligent Drug Systems and Smart Bio-Devices (IDS2B), National Yang Ming Chiao Tung University, Hsinchu City 300193, Taiwan; 10 Department of Physical Medicine and Rehabilitation, Far Eastern Memorial Hospital, New Taipei City 220216, Taiwan; 11 Department of Internal Medicine, China Medical University Hospital, Taichung City 404327, Taiwan; 12 School of Medicine, College of Medicine, Taipei Medical University, Taipei 110301, Taiwan

**Keywords:** high-intensity laser therapy, extracorporeal shockwave therapy, lateral epicondylitis, tennis elbow, lateral elbow tendinopathy, systematic review, meta-analysis

## Abstract

**Purpose:** In this systematic review, we compare the effectiveness of high-intensity laser therapy (HILT) and extracorporeal shockwave therapy (ESWT) in treating lateral elbow tendinopathy (LET). **Methods:** A comprehensive search of PubMed, the Cochrane Library, and EMBASE was conducted from database inception to 23 June 2025 to identify randomized controlled trials (RCTs) comparing the two interventions. The primary outcome was pain intensity (visual analog scale or numeric rating scale). Secondary outcomes included upper-limb disability (qDASH), grip strength (pain-free or maximal), ultrasound-measured common extensor tendon thickness, and safety (adverse events and withdrawals). Two reviewers independently extracted data and assessed methodological quality using the Physiotherapy Evidence Database (PEDro) scale; the certainty of evidence was rated using the Grading of Recommendations Assessment, Development and Evaluation (GRADE) approach. Effects were synthesized as SMD (95% CI) using random- or fixed-effects models based on heterogeneity (I^2^). Significance was set at *p* < 0.05. **Results:** Four RCTs met the inclusion criteria and 169 participants were included. Methodological quality was moderate, with moderate-quality evidence indicating a significant improvement in short-term and medium-term upper-limb function in favor of HILT (SMD = −0.42; 95% CI: −0.73 to −0.12 and SMD = −0.50; 95% CI: −0.94 to −0.06, respectively). Evidence ranging from low to moderate quality showed no significant differences between the HILT and ESWT groups in terms of short-term or medium-term resting pain (SMD = −0.50; 95% CI: −1.15 to 0.16 and SMD = −0.42; 95% CI: −1.06 to 0.22, respectively), short-term or medium-term activity pain (SMD = −0.38; 95% CI: −1.05 to 0.29 and SMD = −0.73; 95% CI: −1.65 to 0.19, respectively), short-term or medium-term grip strength (SMD = 0.24; 95% CI: −0.20 to 0.67 and SMD = 0.20; 95% CI: −0.16 to 0.55, respectively), or short-term or medium-term common extensor tendon thickness (SMD = 0.04; 95% CI: −0.50 to 0.59 and SMD = −0.00; 95% CI: −0.55 to 0.55, respectively). **Conclusions:** HILT appears to offer significant benefits in improving upper-limb function at short-term (<1 month) and medium-term (1–3 months) follow-up. Regarding pain, grip strength, and tendon thickness, the pooled effects did not show clear between-group differences. Evidence certainty ranged from low to moderate, demonstrating that trials with a follow-up period beyond 3 months are needed to evaluate long-term efficacy. **Systematic review registration number:** PROSPERO: CRD420251026387.

## 1. Introduction

Lateral elbow tendinopathy (LET), commonly known as tennis elbow, is an upper-limb musculoskeletal disorder that affects 3% of the general population [1]. The disorder primarily affects individuals aged 40–45 years employed in manual labor occupations, with no sex-based differences [2]. Despite its traditional name, LET is a tendinosis resulting from repetitive gripping and overuse of the common extensor tendon and the extensor carpi radialis brevis [3]. The symptoms of LET are characterized by lateral epicondyle pain exacerbated by wrist extension, elbow pronation, supination movements, and typical movements in some professions [4]. LET often results in disability of the extremities and decreased performance of sport and occupational activities, negatively impacting patients’ quality of life and socio-economic status. Numerous forms of treatment have been proposed to manage the symptoms of LET. Conservative management of LET is commonly delivered by physiotherapists and includes patient education/activity modification, bracing, manual therapy, and structured therapeutic exercise with adjunct physical modalities (e.g., extracorporeal shockwave therapy (ESWT) and low-level laser therapy (LLLT)), with physician-administered options including injections and, in refractory cases, surgical procedures [5,6,7,8,9]. At present, however, no consensus has been reached on the most effective management strategy.

ESWT uses high-intensity acoustic pressure waves to target specific parts of the body and achieves pain relief by destroying unmyelinated sensory fibers, inducing neovascularization stimulation, and boosting collagen synthesis in degenerative tissues [10]. ESWT is generally well tolerated; however, transient local reactions such as skin erythema and ecchymosis have been reported. In one report, these effects were noted in approximately 20% of treated patients [11]. The therapeutic effect of ESWT on LET has been reported with favorable outcomes, including alleviation of pain and improvement of function [12,13]. In a systematic review and network meta-analysis conducted by Liu et al., the authors compared ESWT and five different injection therapies, and the results showed that ESWT is the best treatment option for grip strength recovery [14].

High-intensity laser therapy (HILT) is a new, non-invasive, and painless option, and its therapeutic effects have been reported to include anti-inflammatory, anti-edema, and analgesia [15]. The application of HILT has been proven effective in numerous musculoskeletal diseases, with favorable results [16,17]. The efficacy of HILT in LET has also been investigated, with results showing that it is a reliable, safe, and effective treatment option [18,19]. Patients with LET who received HILT exhibited significant improvement in handgrip strength, pain, disability, and quality of life parameters [18,19].

Although ESWT and HILT have been reported to induce effective and pain-improving results in LET, only a few RCTs have directly compared these two treatment options in LET [4,20,21,22,23]. Through this systematic review and meta-analysis, we therefore aimed to compare the effectiveness of HILT versus ESWT for LET across patient-relevant clinical outcomes, including pain, upper-limb function, grip strength, and ultrasound-measured tendon thickness.

## 2. Materials and Methods

### 2.1. Study Design

This review was performed in accordance with the recommendations of the Cochrane Handbook for Systematic Reviews of Interventions [24] and is reported following the Preferred Reporting Items for Systematic Reviews and Meta-Analyses guidelines [25]. This systematic review was registered in the International Prospective Register of Systematic Reviews database under registration number CRD420251026387 on 5 April 2025.

### 2.2. Eligibility Criteria

This study included RCTs with parallel, pilot, or crossover study designs. With regard to crossover studies, a sufficient washout period was required for inclusion. The patient (P), intervention (I), comparison (C), outcome (O), and study design (S) model was used to identify eligible studies as follows: (P) participants with LET of any severity level; (I) HILT administered as an adjunct to exercise therapy or a standardized rehabilitation program; (C) ESWT administered as an adjunct to the same exercise therapy or standardized rehabilitation program; (O) clinical outcomes, namely, pain, upper-limb function, grip strength, and common extensor tendon thickness at short-term (<1 month), medium-term (1–3 months), and long-term (>3 months) intervals; and (S) RCTs including parallel and crossover designs.

Excluded articles included protocols, non-peer-reviewed articles, conference papers, and letters to the editor. Moreover, we excluded crossover studies that did not include a washout period. No language restriction was applied to this review’s search strategy.

### 2.3. Search Strategy

To identify relevant studies in the literature, a systematic search was conducted across PubMed, EMBASE, and Cochrane Library from their inception to 23 June 2025. The search parameters encompassed terms and synonyms pertaining to LET, HILT, and ESWT; detailed strategies are provided in Appendix A. Where applicable, database filters were employed to isolate RCTs. Furthermore, we manually scrutinized the reference lists of retrieved papers to identify additional eligible studies. Two independent reviewers performed the initial screening of titles and abstracts, resolving any discrepancies through consensus or consultation with a third investigator. Remaining articles underwent a rigorous full-text review to confirm final eligibility.

Two authors extracted data from each study by using a structured form, and the char A standardized form was utilized by two authors to extract data from the selected studies. Captured information included the following: (1) study identifiers (author and year); (2) participant demographics and clinical profiles; (3) therapeutic protocols and follow-up timelines; and (4) specific outcome data. For the experimental and control cohorts, post-intervention means and standard deviations were culled. In cases of missing data, corresponding authors were contacted via email. The primary endpoint was pain intensity, with secondary metrics including upper-limb function, grip strength, and common extensor tendon thickness. Follow-up intervals were categorized as short-term (<1 month), medium-term (1–3 months), or long-term (>3 months).

### 2.4. Methodological Quality Assessment

To evaluate the risk of bias within the selected RCTs, the Physiotherapy Evidence Database (PEDro) scale was employed [26]. This assessment was conducted independently by two researchers, with any inconsistencies resolved through consultation with a third investigator. The total PEDro score (ranging from 0 to 10) was derived by summing the points from items 2 through 11. Specifically, study quality was categorized as poor (<4), fair (4–5), good (6–8), or excellent (9–10) [26]. Crucially, no studies were excluded based on their methodological quality score.

### 2.5. Statistical Analysis

Statistical analyses were performed using RevMan 5.4 software, which is provided by the Cochrane Collaboration (The Cochrane Collaboration, Copenhagen, Denmark) https://training.cochrane.org/online-learning/core-software-cochrane-reviews/revman/revman-5-download (accessed on 26 June 2025)). To account for potential baseline imbalances, change-from-baseline values (the difference between post-intervention and baseline means) were used to calculate the effect estimates. For studies that did not provide the standard deviation of the change (SD_change_), it was imputed using a correlation coefficient of 0.5 in accordance with the Cochrane Handbook for Systematic Reviews of Interventions [24]. Results with *p* < 0.05 were considered statistically significant. In this review, we used the I^2^ test to objectively measure statistical heterogeneity, with I^2^ > 50% indicating significant heterogeneity [27]. A random-effects model was consistently applied to all meta-analyses. This decision was made a priori because the included trials involved diverse treatment protocols, which inherently introduce clinical heterogeneity. While I^2^ was used to quantify statistical heterogeneity, the random-effects model ensures that the pooled SMD reflects the distribution of effects across these varying clinical contexts. Continuous variables are presented as standardized mean differences (SMDs) with 95% confidence intervals (CIs). SMDs calculated using Cohen’s d were employed to measure the probable clinical meaningfulness of the relationships between variables in a population. An SMD < 0.2 indicated a clinically meaningless effect; an SMD in the range of 0.2–0.5 indicated a small effect; an SMD in the range of 0.5–0.8 indicated a moderate effect; and an SMD > 0.8 indicated a large effect [28]. A funnel plot was constructed to examine publication bias if the number of studies included in each analysis was more than 10.

To assess the robustness and stability of our pooled estimates, especially in cases of substantial heterogeneity (I^2^ > 50%), sensitivity analyses were conducted using the leave-one-out method. This process involved sequentially excluding individual studies from each analysis to determine if the significance of the results or the magnitude of heterogeneity was influenced by any single trial.

### 2.6. Evidence Grading

The Grading of Recommendations, Assessment, Development, and Evaluation (GRADE) approach was used to measure the quality of evidence as confidence in effect estimates [29]. This assessment was performed independently by two reviewers. Disagreements regarding the downgrading of evidence were resolved through consensus or consultation with a third author. This method was employed to examine the quality of the publication based on the study design (randomized trials vs. nonrandomized design), risk of bias, inconsistency, imprecision, indirectness, and publication bias; size and trend in the effect are also considered [29].

## 3. Results

**Figure 1:**
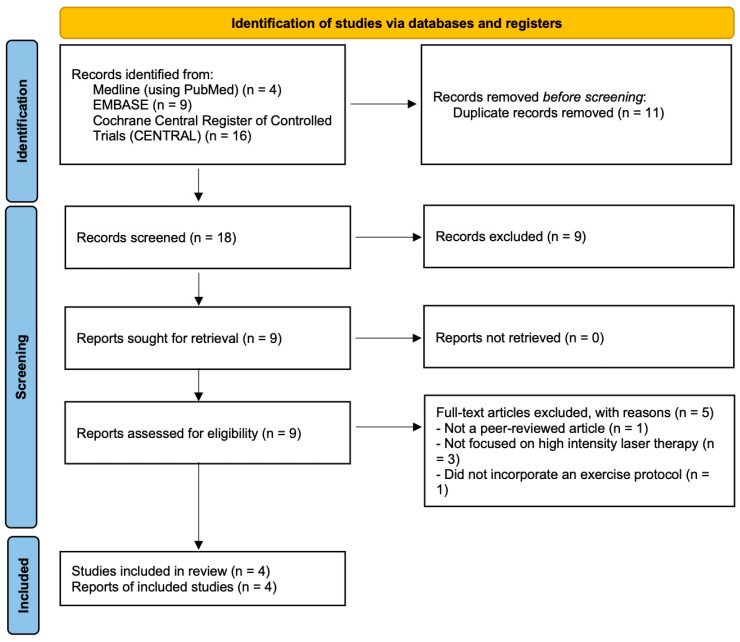
Article selection process.

By using the search terms reported in Appendix A, we initially retrieved 29 studies (4 studies from PubMed, 9 studies from Embase, and 16 from Cochrane Library). Among these studies, 11 duplicates were excluded using EndNote 20 (Version 20; Clarivate, Philadelphia, PA, USA, 2020) [30]. Furthermore, nine studies that did not meet the inclusion criteria were excluded after their titles and abstracts were screened. We screened the full texts of the remaining nine articles and determined that five studies did not fulfill the inclusion and exclusion criteria (one was not a peer-reviewed article, three did not focus on high-intensity laser therapy, and one did not incorporate an exercise protocol). The study by Kang et al. did not incorporate an exercise protocol and was thus not included in the analysis [22]. Ultimately, four studies were included in this study [4,20,21,23]. The comprehensive search was updated through 23 June 2025. However, no eligible RCTs published in the year 2025 were identified at the time of the final search. Consequently, all studies included in this review were published between 2022 and 2024 [4,20,21,23]. A flowchart showing the article selection process is presented in Figure 1.

The meta-analysis included a total of 169 participants across the analyzed studies, with 84 in the intervention (HILT) group and 85 in the control (ESWT) group. All of the selected studies were RCTs [4,20,21,23]. Regarding the outcomes, four studies reported pain scale [4,20,21,23], four reported upper-limb function [4,20,21,23], four reported grip strength [4,20,21,23], and two reported common extensor thickness [21,23]. Although crossover trials were eligible based on our inclusion criteria, no such studies met the eligibility requirements during the screening process. Consequently, all four studies ultimately included in this review followed a parallel-group design. The characteristics of the included studies are listed in Table 1. The HILT and ESWT protocols and dosing parameters are listed in Table 2 and Table 3.

Two reviewers independently evaluated the methodological quality of the included RCTs by using a PEDro scale [26]. One study received a rating of 7 [21], whereas three received a rating of 6 [4,20,23], all of which were considered “good”. The methodological quality of the articles is summarized in Table 4.

### 3.1. Short-Term Resting Pain

**Figure 2:**
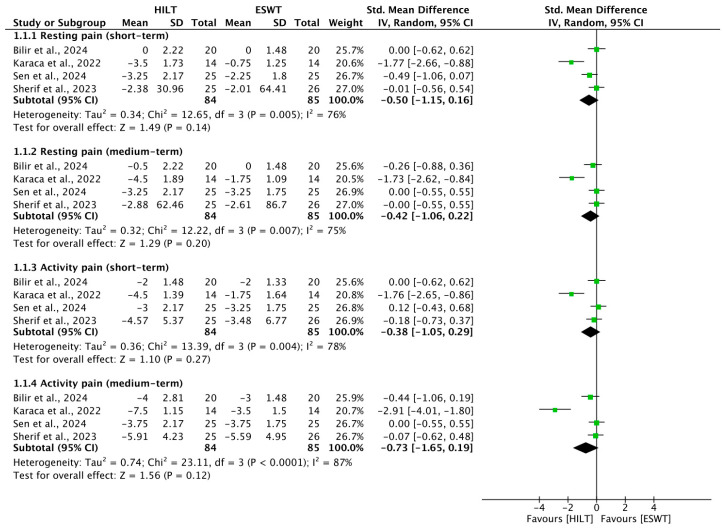
Forest plot of the results of the pain scale. SD, standard deviation; 95% CI, 95% confident interval; HILT, high-intensity laser therapy; ESWT, extracorporeal shockwave therapy. Included studies: Bilir et al., 2024 [21]; Karaca et al., 2022 [4]; Sen et al., 2024 [20]; Sherif et al., 2023 [23].

Four RCTs [4,20,21,23] assessed resting pain levels in the short term, involving a total of 169 participants (84 HILT; 85 ESWT). Significant inter-study heterogeneity was detected (I^2^ = 76%, *p* = 0.005). The pooled analysis indicated that HILT did not provide a statistically superior reduction in resting pain compared to ESWT (SMD = −0.50; 95% CI: −1.15 to 0.16; *p* = 0.14) (Figure 2).

### 3.2. Medium-Term Resting Pain

For the medium-term follow-up, resting pain outcomes were reported in four trials [4,20,21,23]. Similarly to the short-term findings, substantial heterogeneity was observed (I^2^ = 75%, *p* = 0.007). The results showed no significant difference between the two therapeutic modalities (SMD = −0.42; 95% CI: −1.06 to 0.22) (Figure 2).

### 3.3. Short-Term Activity Pain

Activity-induced pain in the short term was investigated in four studies [4,20,21,23]. A high degree of heterogeneity was found (I^2^ = 78%, *p* = 0.004). While HILT showed a trend toward better pain relief during activity, the difference did not reach statistical significance (SMD = −0.38; 95% CI: −1.05 to 0.29) (Figure 2).

### 3.4. Medium-Term Activity Pain

Activity-induced pain in the medium term was investigated in four studies [4,20,21,23]. A high degree of heterogeneity was found (I^2^ = 87%, *p* < 0.0001). The results showed no significant difference between the two therapeutic modalities (SMD = −0.73; 95% CI: −1.65 to 0.19) (Figure 2).

### 3.5. Short-Term Upper-Limb Function

**Figure 3:**
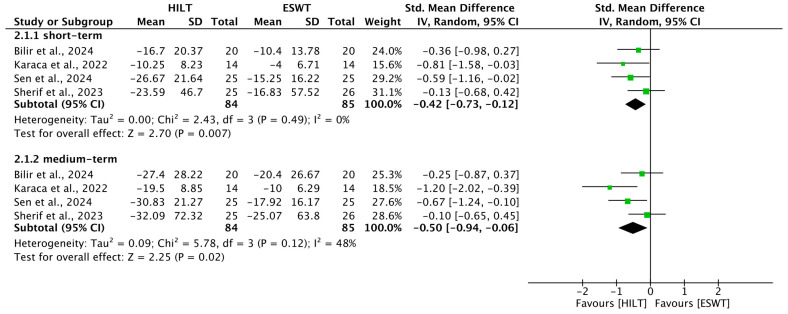
Forest plot of the results of upper-limb function. SD, standard deviation; 95% CI, 95% confident interval; HILT, high-intensity laser therapy; ESWT, extracorporeal shockwave therapy. Included studies: Bilir et al., 2024 [21]; Karaca et al., 2022 [4]; Sen et al., 2024 [20]; Sherif et al., 2023 [23].

Upper-extremity functional status in the short term was reported in four trials [4,20,21,23]. Heterogeneity remained very low (I^2^ = 0%, *p* = 0.49). The synthesized data favored HILT, showing a statistically significant improvement in functional outcomes (SMD = −0.42; 95% CI: −0.73 to −0.12) (Figure 3).

### 3.6. Medium-Term Upper-Limb Function

Four studies provided data on medium-term upper-limb disability [4,20,21,23]. With moderate statistical heterogeneity (I^2^ = 48%, *p* = 0.12), HILT again showed superior functional recovery compared to the ESWT group (SMD = −0.50; 95% CI: −0.94 to −0.06) (Figure 3).

### 3.7. Short-Term Grip Strength

**Figure 4:**
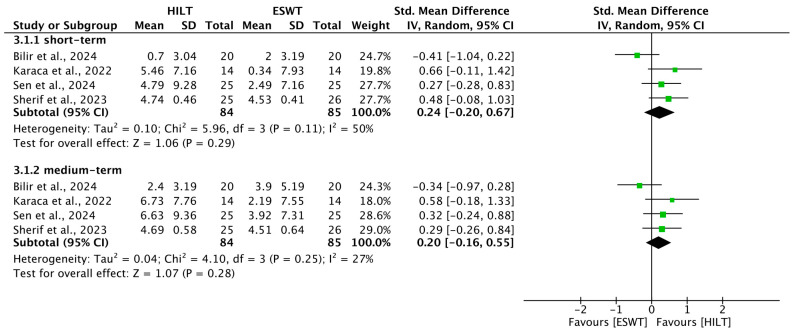
Forest plot of the results of grip strength. SD, standard deviation; 95% CI, 95% confident interval; HILT, high-intensity laser therapy; ESWT, extracorporeal shockwave therapy. Included studies: Bilir et al., 2024 [21]; Karaca et al., 2022 [4]; Sen et al., 2024 [20]; Sherif et al., 2023 [23].

Regarding grip strength in the short term, data from four trials were pooled [4,20,21,23]. Heterogeneity was moderate (I^2^ = 50%, *p* = 0.11). The analysis showed no significant evidence that one treatment was superior to the other (SMD = 0.24; 95% CI: −0.20 to 0.67) (Figure 4).

### 3.8. Medium-Term Grip Strength

Medium-term grip strength recovery was consistent across four studies with low detected heterogeneity (I^2^ = 27%, *p* = 0.25) [4,20,21,23]. No statistically significant difference was found between the HILT and ESWT groups (SMD = 0.20; 95% CI: −0.16 to 0.55) (Figure 4).

### 3.9. Short-Term Common Extensor Tendon Thickness

**Figure 5:**
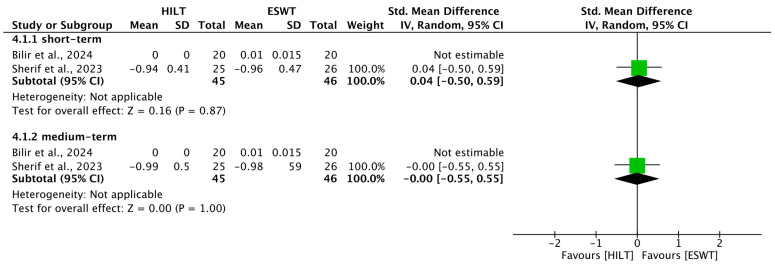
Forest plot of the results of common extensor tendon thickness. SD, standard deviation; 95% CI, 95% confident interval; HILT, high-intensity laser therapy; ESWT, extracorporeal shockwave therapy. Included studies: Bilir et al., 2024 [21]; Sherif et al., 2023 [23].

In two studies, sonographic changes in common extensor tendon thickness were reported shortly after treatment [21,23]. Heterogeneity was not applicable. The comparative effect on tendon thickness was not statistically significant between groups (SMD = 0.04; 95% CI: −0.50 to 0.59) (Figure 5).

### 3.10. Medium-Term Common Extensor Tendon Thickness

Two trials assessed common extensor tendon thickness at a medium-term interval [21,23]. Heterogeneity was not applicable. No significant disparity was observed between HILT and ESWT regarding this morphological outcome (SMD = −0.00; 95% CI: −0.55 to 0.55) (Figure 5).

### 3.11. Sensitivity Analysis

To investigate the source of high heterogeneity observed in pain-related outcomes, a leave-one-out sensitivity analysis was performed. In the analysis, the study by Karaca et al. [4] was identified as the primary contributor to statistical variance. Upon its systematic exclusion, the heterogeneity for short-term and medium-term resting pain, in addition to activity pain at both time points, was reduced to 0%. Notably, within this homogeneous subset, the pooled effect estimates for these pain parameters did not reach statistical significance.

### 3.12. Quality of Evidence

The quality of evidence was assessed using the GRADE approach. Overall, the quality of evidence for all outcomes was rated as low to moderate, likely due to the risk of bias and heterogeneity among the included studies. Detailed assessments of the quality of evidence are presented in Table 5.

While our methodology pre-defined long-term follow-up as periods exceeding 3 months, none of the included RCTs provided outcome data beyond this timeframe. Consequently, long-term effectiveness could not be analyzed in this meta-analysis.

## 4. Discussion

### 4.1. Main Findings of the Review

Across four RCTs, HILT showed a statistically significant advantage over ESWT for upper-limb disability in the short and medium term with low heterogeneity. Regarding resting pain, activity pain, grip strength, and CET thickness, there were no significant between-group differences at the short- or medium-term follow-up. Overall certainty based on GRADE ranged from low to moderate. Potential contributors to heterogeneity included trials that converted medians/IQRs to means/SDs.

### 4.2. Evidence-Based Perspectives on ESWT and HILT Modalities

ESWT alleviates musculoskeletal pain through several mechanisms, including stimulation of nerve receptors, reduced nerve sensitivity, modulation of nociceptor activity, promotion of neovascularization, and increased collagen synthesis in degenerative tissue [10]. The results of previous studies have shown that ESWT offers superior outcomes in terms of pain relief, functional recovery, and grip strength when compared with other conservative interventions for LET [31,32]. For instance, Rompe et al. [13] conducted a placebo-controlled trial involving 78 tennis players with chronic LET (≥12 months) and found significantly greater improvements in pain and upper-extremity function in the ESWT group than in the placebo group [13]. Similarly, the results of a network meta-analysis by Liu et al. [14] demonstrated that ESWT was the most effective treatment for restoring grip strength compared to five different injection therapies.

There are two main types of ESWT: focused and radial. Focused ESWT delivers concentrated energy to deeper tissues; in comparison, radial ESWT disperses energy more broadly and superficially. Both have demonstrated efficacy in musculoskeletal conditions; however, a consensus on their relative effectiveness remains absent. The results of a systematic review by Soon et al. [33], which included 12 studies with a total of 1104 participants, demonstrated that although radial ESWT showed statistically greater improvements in pain reduction and grip strength, the clinical significance of these findings was limited [33].

Laser-based therapies such as HILT and low-level laser therapy (LLLT) offer non-invasive, painless options for managing musculoskeletal disorders. Both operate in the near-infrared spectrum with similar tissue penetration depths, primarily targeting superficial layers [16]. HILT, with its higher power output (over 0.5 W), provides broader coverage and produces photothermal effects [34,35].

HILT is believed to exert therapeutic effects through enhanced metabolism, beta-endorphin release, and photochemical and photothermal actions that confer anti-inflammatory, anti-edematous, and analgesic benefits [15]. Its application in various musculoskeletal disorders has shown promising results in reducing pain and improving function [16,17,36]. For LET specifically, studies have reported that HILT significantly improves grip strength, pain scores, functional capacity, and quality of life in both short- and long-term follow-ups [18,19]. In two recent systematic reviews with meta-analyses, the authors synthesized the evidence for HILT in lateral epicondylitis. ElMeligie et al. [37] pooled six randomized trials (*n* = 344) comparing HILT to control for other modalities and found small improvements in pain with low-quality evidence, combined with an improvement in the SF-36 physical component; by contrast, grip strength and hand function did not differ significantly from comparators. Tang et al. [38] likewise examined six studies (*n* = 321), reporting that HILT reduced pain versus active controls and improved quality of life in the physical domain, with effects on disability and grip strength not being significant; the authors graded the overall body of evidence as weak and noted heterogeneity and risks of selection, performance, and detection bias. Importantly, in these reviews, the authors addressed whether HILT is effective versus control or assorted non-ESWT comparators; they did not resolve the common clinical decision between HILT and ESWT.

### 4.3. Comparative Advantages of Each Intervention

This review is designed to fill the current gap in the literature by providing direct, comparative estimates of the effect of HILT vs. ESWT on pain, disability (qDASH), grip strength, and tendon thickness. In this review, we found that HILT showed superior outcomes in upper-limb function compared to ESWT up to three months post-treatment. This finding suggests that HILT may exert more sustained therapeutic effects. The slow absorption of light by chromophores during HILT leads to ATP production and enhanced tissue regeneration, potentially contributing to its longer-lasting benefits [39]. Akkurt et al. [18] studied long-term HILT effects in 30 patients with LET (37 elbows) and reported progressive improvements in pain (both at rest and during activity), upper-limb function, and grip strength starting at two weeks and continuing up to six months post-treatment [18]. Comparable long-term effects were observed with ESWT. For instance, in a clinical trial by Pettrone et al. [40] involving 114 LET patients, it was found that 61% of participants in the ESWT group experienced at least a 50% improvement in pain scores compared to the placebo, with the effects lasting up to one year.

Increased CET thickness has been associated with LET. Lee et al. [41] evaluated 48 LET patients and 68 healthy controls using ultrasound, with significantly thicker CET being found in the LET group. CET thickness greater than 4.2 mm and area ≥ 32 mm^2^ were strongly predictive of LET [41]. Özmen et al. [42] investigated treatment effects on CET thickness using ESWT, kinesio taping, and ultrasound therapy. While all treatments reduced pain, only the ESWT group showed reduced CET thickness [42]. Bilir et al. [21] reported significant reductions in CET thickness in both HILT and ESWT groups at 1 and 6 weeks post-treatment. Similarly, Thammajaree et al. [43] demonstrated that both radial ESWT and HILT reduced plantar fascia thickness and pain in patients with plantar fasciitis after 3 weeks of treatment. Given the correlation between tendon thickness and LET, the authors of future studies should consider CET thickness as a potential outcome measure in evaluating treatment efficacy.

### 4.4. Differences in Energy Dosing

The authors of the included studies used heterogeneous dosing and device parameters. HILT protocols ranged from staged analgesic/biostimulation settings to higher-fluence, pulsed regimens; reported energy per session varied by trial. ESWT protocols differed by pulse number, frequency, pressure (bar), and energy flux density (mJ/mm^2^) or device type (focused vs. radial). Detailed protocols and dosing parameters for both HILT and ESWT are compiled in Table 2 and Table 3 of this manuscript and were extracted verbatim from the trials. This variability likely contributed to between-study heterogeneity.

Notably, there is no universally accepted dosing guideline for HILT; existing World Association for Laser Therapy (WALT) [44] recommendations pertain primarily to LLLT and are not directly applicable to HILT. In the WALT dose table for 780–860 nm GaAlAs lasers (continuous or pulsed; mean output 5–500 mW), the recommended dose of LLLT for LET is 4 J applied to 1–2 points (or cm^2^), with a maximum irradiance of 100 mW/cm^2^.

By contrast, ESWT has condition-specific guidance in LET (e.g., International Society for Medical Shockwave Treatment [45] recommendations). For radial ESWT, the International Society for Medical Shockwave Treatment guideline summarizes typical parameter ranges as 1.4–2.5 bar, delivered over 3–5 sessions, with a treatment frequency up to 8 Hz and approximately 2000 pulses per session. For LET, establishing transparent, harmonized dosing templates (e.g., wavelength/power/fluence for HILT) would likely reduce heterogeneity and improve interpretability.

### 4.5. Clinical Relevance of Findings

To the best of our knowledge, there are few systematic reviews and meta-analyses that directly compare the effectiveness of HILT and ESWT in treating LET. For adults with LET, the available evidence suggests that HILT may be preferred when rapid functional improvement and activity-related pain reduction are clinical priorities; in comparison, ESWT remains a reasonable alternative when aiming for pain control, grip strength recovery, or tendon morphologic outcomes comparable to HILT. In light of the limited number of trials, variability in dosing protocols, and relatively short follow-up, future well-designed RCTs with standardized reporting and patient-centered outcomes are needed to confirm clinical importance and durability of effects. In practice, both HILT and ESWT should be considered adjunctive modalities within a broader rehabilitation approach rather than stand-alone treatments. Selection can be guided by availability, cost, time burden (number of sessions), patient preference, contraindications, and tolerance, with shared decision-making emphasizing functional goals and activity-related pain.

### 4.6. Study Limitations

Limitations of this review include the novelty of the research question, which precluded direct comparisons with the existing literature, and the limited number of available RCTs comparing HILT and ESWT specifically in LET. Furthermore, a significant constraint is the lack of long-term outcome data; all included studies reported results only up to a medium-term follow-up of three months, thus restricting the generalizability and strength of the conclusions, particularly regarding the durability of treatment effects for a chronic condition such as LET.

## 5. Conclusions

In this review, our findings show that HILT offers better upper-limb function improvement than ESWT at short-term follow-up (<1 month) and medium-term follow-up (1–3 months). Both treatments were effective in improving resting pain, activity pain, grip strength, and CET thickness, with no clear superiority of one over the other at either time point. In light of the limited number of studies and low- to moderate-quality evidence, the findings should be interpreted with caution. Because none of the trials provided long-term outcomes (defined as >3 months), future studies should incorporate long-term follow-up beyond three months to evaluate the long-term efficacy of HILT and ESWT.

## Figures and Tables

**Table 1:** Study characteristics.

Author, Year	Intervention Group								Follow-Up Timing	Outcome Measurement
		Number of Patients	Participant Gender	Age (y), Mean (SD)	Intervention Protocol	Additional Treatments	Adverse Events	Dropout Rates		
Bilir et al., 2024 [21]	Experimental group	20	Female = 11 Male = 9	44.35 (7.71)	HILT Protocol: Total of 9 sessions over 3 weeks (3 sessions/week). Analgesic Phase: 75 s at 4 W (6 J/cm^2^) using circular motions. Biostimulation Phase: 12.5 min at 6 W (100–150 J/cm^2^) using linear motions.	Core Exercise Program (Both Groups): A combination of flexibility, stretching, and muscle-strengthening exercises. Exercise Dosage: 3 sets of 10 repetitions per session, with a 1 min rest interval.	-	4/24 = 16.7%	1 and 6 weeks after the intervention	Pain intensity (VAS) (Resting and Activity Pain), upper-limb disability (qDASH), grip strength (dynamometer), common extensor tendon thickness (ultrasound-measured)
	Control group	20	Female = 9 Male = 11	45.80 (11.65)	ESWT Protocol: Total of 3 sessions (once weekly). Parameters: 2000 pulses at 10 Hz and 2.5 bar per session.		-	3/23 = 13.0%		
Sherif et al., 2023 [23]	Experimental group	25	Female = 13 Male = 12	40.06 (1.88)	HILT Protocol: Total of 9 sessions over 3 weeks (3 sessions/week). Analgesic Phase: 75 s at 8 W (6 J/cm^2^) in an intermittent phase. Biostimulation Phase: 30 s at 6 W (120–150 J/cm^2^) in a continuous phase.	Core Exercise Program (Both Groups): ROM exercises, stretching, and strengthening (specifically handgrip strength). Exercise Dosage: 3 sets of 10 repetitions daily with 1 min breaks.	-	-	At the end of the intervention and 6 weeks after the intervention	Pain intensity (VAS) (Resting and Activity Pain), upper-limb disability (qDASH), grip strength (dynamometer), common extensor tendon thickness (ultrasound-measured)
	Control group	26	Female = 13 Male = 13	41.86 (8.43)	ESWT Protocol: Total of 3 sessions (once weekly). Parameters: 2000 pulses at 10 Hz and 2.5 bar per session.		-	-		
Karaca et al., 2022 [4]	Experimental group	14	Female = 9 Male = 5	35 (24–55) (median (range))	HILT Protocol: Total of 4 sessions over 2 weeks (2 sessions/week). Analgesic Phase: 75 s at 4 W (6 J/cm^2^). Biostimulation Phase: 12.5 min at 6 W (12 J/cm^2^).	Core Exercise Program (Both Groups): Home-based eccentric strengthening and forearm stretching. Exercise Dosage: 10 repetitions, 3 times daily for 4 weeks. Additional PT: Cold packs (15 min), continuous ultrasound (5 min), and TENS (30 min).	-	-	2 and 6 weeks after the intervention	Pain intensity (VAS) (Resting and Activity Pain), grip strength (dynamometer), function level (Patient-Rated Tennis Elbow Evaluation questionnaire)
	Control group	14	Female = 7 Male = 7	34 (22–52) (median (range))	ESWT Protocol: Total of 4 sessions over 2 weeks (2 sessions/week). Parameters: 2000 pulses at 10 Hz and 2.5 bar per session.		-	-		
Sen et al., 2024 [20]	Experimental group	25	Female = 13 Male = 12	45.40 (8.54)	HILT Protocol: Total of 15 consecutive sessions completed within 3 weeks. Parameters: 15 W power, 15 Hz frequency in pulsed mode, delivering 39 J/cm^2^ per point to the most painful area.	Core Exercise Program (Both Groups): Forearm rehabilitation consisting of stretching and strengthening exercises. Exercise Dosage: Exercises were performed regularly under guidance to complement the primary modalities.	-	5/30 = 16.7%	3 and 12 weeks after the intervention	Pain intensity (VAS) (Resting and Activity Pain), upper-limb disability (qDASH), grip strength (dynamometer)
	Control group	25	Female = 16 Male = 9	47.08 (6.05)	ESWT Protocol: Total of 3 sessions administered once weekly for 3 weeks. Parameters: Radial ESWT providing 1500 pulses per session at a frequency of 8 Hz and energy flux density of 0.18 mJ/mm^2^.		-	5/30 = 16.7%		

SD, standard deviation; HILT, high-intensity laser therapy; ESWT, extracorporeal shockwave therapy; qDASH, quick Disabilities of Arm, Shoulder, and Hand.

**Table 2:** HILT parameters.

	Treatment Protocol	Mode	Power	Average Power (W)	Fluence (J/cm^2^)	Energy Dose (J)/Session	Spot Diameter (mm)
Bilir et al., 2024 [21]	First 3 sessions at an analgesic dose; subsequent 6 sessions at a biostimulation dose; 3 sessions/week, total 9 sessions	Not reported	4 W (first 3 sessions); 6 W (last 6 sessions)	Not reported	6 J/cm^2^ (early phase); 100–150 J/cm^2^ (later phase)	Analgesic sessions: 4 W × 75 s = 300 J per session Biostimulation sessions: 6 W × 12 min 30 s = 6 W × 750 s = 4500 J per session	Not reported
Sherif et al., 2023 [23]	First 3 sessions of 75 s, 8 W, 6 J/cm^2^; next 6 sessions of 30 s, 6 W, 120–150 J/cm^2^; total 9 sessions, 3/week for 3 weeks	Intermittent → continuous	8 W (first phase); 6 W (second phase)	Not reported	6 J/cm^2^; and 120–150 J/cm^2^	Total energy 150 J for the first phase	Not reported
Karaca et al., 2022 [4]	Analgesic phase 4 W/6 J/cm^2^ for 75 s, followed by biostimulation phase 6 W/12 J/cm^2^ for 12 min 30 s; 2 sessions/week, total 4 sessions	Not reported	4 W; 6 W	Not reported	6 J/cm^2^; 12 J/cm^2^	4 W × 75 s = 300 J + 6 W × 750 s = 4500 J ⇒ 4800 J per session	Spot area 0.3 cm^2^ (Diameter ≈ 6.18 mm)
Sen et al., 2024 [20]	15 consecutive sessions completed within 3 weeks	Pulsed	15 W	15 W	39 J/cm^2^	Not reported	Not reported

HILT: high-intensity laser therapy.

**Table 3:** ESWT parameters.

	Treatment Protocol	ESWT Type	Energy Density	Pressure	Frequency
Bilir et al., 2024 [21]	1 session/week, total 3 sessions	Not reported	Not reported	2.5 bar	10 Hz, 2000 pulses
Sherif et al., 2023 [23]	1 session/week, total 3 sessions	Not reported	Not reported	2.5 bar	10 Hz, 2000 pulses
Karaca et al., 2022 [4]	2 sessions/week, total 4 sessions	Not reported	Not reported	2.5 bar	10 Hz, 2000 pulses
Sen et al., 2024 [20]	1 session/week for 3 weeks	Radial	0.18 mJ/mm^2^	Not reported	1500 pulses/session

ESWT: extracorporeal shockwave therapy.

**Table 4:** PEDro scale.

	1 *	2	3	4	5	6	7	8	9	10	11	Total
Bilir et al., 2024 [21]	Y	Y	N	Y	N	N	Y	Y	Y	Y	Y	7
Sherif et al., 2023 [23]	Y	Y	N	Y	N	N	N	Y	Y	Y	Y	6
Karaca et al., 2022 [4]	Y	Y	N	Y	N	N	N	Y	Y	Y	Y	6
Sen et al., 2024 [20]	Y	Y	N	Y	N	N	N	Y	Y	Y	Y	6

PEDro scale criteria: 1, eligibility criteria and source of participants; 2, random allocation; 3, concealed allocation; 4, baseline comparability; 5, blinded participants; 6, blinded therapists; 7, blind assessors; 8, adequate follow-up; 9, intention-to-treat analysis; 10, between-group comparisons; 11, point estimates and variability. * Not included in the calculation of the total score.

**Table 5:** GRADE assessment.

Certainty Assessment							Number of Patients		Effect		Certainty	Importance
Number of Studies	Study Design	Risk of Bias	Inconsistency	Indirectness	Imprecision	Other Considerations	Experimental Group	Control Group	Relative (95% CI)	Absolute (95% CI)		
Short-Term Resting Pain												
4	randomized controlled trials	Serious ^a^	serious ^b^	not serious	not serious	none	84	85	-	SMD 0.05 lower (0.16 lower to 1.15 higher)	⨁⨁◯◯ Low ^a,b^	Important
Medium-Term Resting Pain												
4	randomized controlled trials	Serious ^a^	serious ^b^	not serious	not serious	none	84	85	-	SMD 0.42 lower (0.22 lower to 1.06 higher)	⨁⨁◯◯ Low ^a,b^	Important
Short-Term Activity Pain												
4	randomized controlled trials	Serious ^a^	serious ^b^	not serious	not serious	none	84	85	-	SMD 0.38 lower (0.29 lower to 1.05 higher)	⨁⨁◯◯ Low ^a,b^	Important
Medium-Term Activity Pain												
4	randomized controlled trials	Serious ^a^	serious ^b^	not serious	not serious	none	84	85	-	SMD 0.73 lower (0.19 lower to 1.65 higher)	⨁⨁◯◯ Low ^a,b^	Important
Short-Term Upper-Limb Function												
4	randomized controlled trials	Serious ^a^	not serious	not serious	not serious	none	84	85	-	SMD 0.42 lower (0.12 lower to 0.73 higher)	⨁⨁⨁◯ Moderate ^a^	Important
Medium-Term Upper-Limb Function												
4	randomized controlled trials	Serious ^a^	not serious	not serious	not serious	none	84	85	-	SMD 0.50 lower (0.06 lower to 0.94 higher)	⨁⨁⨁◯ Moderate ^a^	Important
Short-Term Grip Strength												
4	randomized controlled trials	Serious ^a^	not serious	not serious	not serious	none	84	85	-	SMD 0.24 lower (0.20 lower to 0.67 higher)	⨁⨁⨁◯ Moderate ^a^	Important
Medium-Term Grip Strength												
4	randomized controlled trials	Serious ^a^	not serious	not serious	not serious	none	84	85	-	SMD 0.20 lower (0.16 lower to 0.55 higher)	⨁⨁⨁◯ Moderate ^a^	Important
Short-Term Common Extensor Tendon Thickness												
2	randomized controlled trials	Serious ^a^	not serious	not serious	not serious	none	45	46	-	SMD 0.04 lower (0.50 lower to 0.59 higher)	⨁⨁⨁◯ Moderate ^a^	Important
Medium-Term Common Extensor Tendon Thickness												
2	randomized controlled trials	Serious ^a^	not serious	not serious	not serious	none	45	46	-	SMD 0.00 lower (0.55 lower to 0.55 higher)	⨁⨁⨁◯ Moderate ^a^	Important

CI: confidence interval; SMD: standardized mean difference. Explanations: ^a^. lack of blinding; ^b^. high heterogeneity. **Certainty of evidence (GRADE):** ⨁⨁⨁⨁ High; ⨁⨁⨁◯ Moderate; ⨁⨁◯◯ Low; ⨁◯◯◯ Very low.

## Data Availability

All of the data generated or analyzed in this study are included in this published article.

## Abbreviations

The following abbreviations are used in this manuscript:

VAS	Visual Analog Scale
SD	Standard Deviation
CI	Confidence Interval
CET	Common Extensor Tendon
GRADE	Grading of Recommendations Assessment, Development and Evaluation
HILT	High-Intensity Laser Therapy
PEDro	Physiotherapy Evidence Database
LET	Lateral Elbow Tendinopathy
PRISMA	Preferred Reporting Items for Systematic Reviews and Meta-Analyses
RCT	Randomized Controlled Trial
qDASH	Quick Disabilities of the Arm, Shoulder, and Hand
SMD	Standardized Mean Difference
PRTEET	Patient-Rated Tennis Elbow Evaluation—Turkish version

## Appendix A

Keywords used for identifying relevant articles in various electronic databases:

Database	Search Terms
PubMed (inception: 1782)	Search for articles published from database inception to 6 April 2025.
#1	Shockwave
#2	Shock wave
#3	ESWT
#4	Extracorporeal Shockwave Therapy [MeSH]
#5	#1 OR #2 OR #3 OR #4
#6	laser
#7	Laser therapy [MeSH]
#8	#6 OR #7
#9	elbow
#10	Epicondyle *
#11	Tennis Elbow [MeSH]
#12	#9 OR #10 OR #11
#13	#5 AND #8 AND #12
Filter	Randomized controlled trial
**Note: Truncation using an asterisk (*) was applied in the search.**	

Database	Search Terms
EMBASE (inception: 1947)	Search for articles published from database inception to 6 April 2025.
#1	Shockwave
#2	Shock wave
#3	ESWT
#4	Extracorporeal Shockwave Therapy/exp
#5	#1 OR #2 OR #3 OR #4
#6	laser
#7	Laser therapy/exp
#8	#6 OR #7
#9	elbow
#10	Epicondyle *
#11	Tennis Elbow/exp
#12	#9 OR #10 OR #11
#13	#5 AND #8 AND #12
Filter	Randomized controlled trial
**Note: Truncation using an asterisk (*) was applied in the search.**	

Database	Search Terms
Cochrane Library (inception: 1971)	Search for articles published from database inception to 6 April 2025.
#1	Shockwave
#2	Shock wave
#3	ESWT
#4	[Extracorporeal Shockwave Therapy] explode all trees
#5	#1 OR #2 OR #3 OR #4
#6	laser
#7	[Laser therapy] explode all trees
#8	#6 OR #7
#9	elbow
#10	Epicondyle *
#11	[Tennis Elbow] explode all trees
#12	#9 OR #10 OR #11
#13	#5 AND #8 AND #12
#14	Random *
#15	#13 AND #14
**Note: Truncation using an asterisk (*) was applied in the search.**	
